# Proposals for person‐centred care in the COVID‐19 era. Delphi study

**DOI:** 10.1111/hex.13218

**Published:** 2021-02-27

**Authors:** José J. Mira, Martín Blanco, Kamila Cheikh‐Moussa, Olga Solas, Aquilino Alonso, Rodrigo Gutierrez, Celia Gómez, Mercedes Guilabert

**Affiliations:** ^1^ Departamento de Salud Alicante‐Sant Joan Grupo Atenea FISABIO Alicante Spain; ^2^ Universidad Miguel Hernández Elche Alicante Spain; ^3^ REDISSEC Alicante Spain; ^4^ Consultor en ámbito sanitario y de la sostenibilidad justa Madrid Spain; ^5^ Consultora en políticas públicas Toledo Spain; ^6^ Área de Servicios Públicos Supramunicipales Diputación Provincial de Sevilla Sevilla Spain; ^7^ Sociedad Española de Atención al Usuario de la Sanidad Barcelona Spain; ^8^ Servicio Cántabro de Salud Santander Spain

**Keywords:** delivery of health care, integrated, health personnel, nurse‐patient relations, patient participation, patient‐centred care, physician‐patient relations, primary health care, spanish health system

## Abstract

**Background:**

In this COVID‐19 era, we need to rethink the criteria used to measure the results of person‐centred care strategies.

**Objective:**

To identify priorities, and criteria that health services can use to pursue actually the goal of achieving person‐centred care.

**Design:**

Three‐phase online qualitative study performed during May–July of 2020 using the Delphi technique.

**Setting and Participants:**

An online platform was used for a consensus meeting of 114 participants, including health planning experts, health‐care institution managers, clinicians and patients.

**Main Outcome Measures:**

Criteria and indicators for the achievement of person‐centred care.

**Main Results:**

The first round began with 125 proposals and 11 dimensions. After the second round, 28 ideas reached a high level of consensus among the participants. Ultimately, the workgroup agreed on 20 criteria for goals in the implementation of person‐centred care during the COVID‐19 era and 21 related indicators to measure goal achievement.

**Discussion:**

Nine dimensions and 28 priorities were identified. These priorities are also in accordance with the quadruple aim approach, which emphasizes the need for care for health‐care professionals, without whom it is impossible to achieve a better quality of care.

**Conclusions:**

Person‐centred care continues to be a key objective. However, new metrics are needed to ensure its continued development during the restoration of public health services beyond the control of COVID‐19.

**Patient or Public Contribution:**

Twelve professionals and patient representatives participated voluntarily in the construction of the baseline questionnaire and in the selection of the criteria and indicators using an online platform for consensus meetings.

## 1 INTRODUCTION

Person‐centred care (PCC) is defined as being respectful and responsive to patient needs and preferences and allowing the patient's values to guide all clinical decisions.^1^ This definition includes respect for the patient's personal rights and active patient participation in the decisions that are made regarding their care.^1^, ^2^

PCC is characterized by an integrative approach aimed at preserving dignity, considering the patients' needs and preferences, improving quality of life and wellbeing, and promoting active patient participation to achieve better health outcomes.^2^, ^3^ Person‐centred goal setting represents a shift from provider‐ or policy‐oriented goals to the inclusion of the patient's personal goals in shared care planning.^4^, ^5^

The PCC model includes integrated care that promotes personal well‐being, active patient participation and co‐planning for better outcomes and considers patient experience as a measurable outcome that can be used to guide changes in resources or procedures at the organisational level.^6^, ^7^, ^8^, ^9^, ^10^ Barriers that could hinder the achievement of PCC goals include resistance to change (including the patient's resistance to participate in joint decision making with their health‐care professionals), lack of professional facilities and limited access to information, which are usually designed using a paternalistic care approach.^11^ Patients and citizens have a lot to say about the scope of PCC; however, directive personnel and staff attitudes are more of a determinant of the paradigm change that is necessary to promote PCC.^4^ Moreover, advances in the evaluation of outcomes from the patient's perspective could be made by, for example, analysing both patient and professional attitudes and incorporating measures using the Patient Reported Experience Measure and the Patient Reported Outcome Measure questionnaires.^12^, ^13^, ^14^

The COVID‐19 pandemic has changed many aspects of the framework for patient‐health‐care professional relationships.^15^ In previous times of crisis, the professional perspective was that the patient experience may need to be sacrificed in the interest of clinical effectiveness.^16^, ^17^ COVID‐19 has introduced new health‐care scenarios and conditions of human interaction and has altered procedures that were established long ago, resulting in unexpected consequences for health‐care equity, accessibility, patient participation in the decision‐making process and the evaluation of results.^12^ These changes in the era of COVID‐19 have made it difficult for more PCC and have required the establishment of new priorities to ensure that the efforts made to implement PCC do not stop. Numerous changes can be seen in European and local scenarios^13^, ^14^ where primary care consists of universal coverage and is the gateway to the health‐care system, with selective attention primarily based on disease type. The pandemic has caused personal care restrictions, and specifically for chronically ill patients, follow‐up that is limited to phone calls. It has also lead to the breakdown of the multidisciplinary structure of primary care teams, due to the need to prioritize staff based on hospital needs and the high demand for emergency and specialized hospital care and infectious disease protocols. These changes highlight the need for the quick set‐up of pending improvements to local health‐care systems and for direct communication between primary and hospital care by incorporating information and communication technologies, and through the implementation of mobile patient monitoring equipment in the health care and follow‐up of chronically ill patients.^14^ Institutions such as the Spanish Society of Family and Community Medicine have presented a series of organisational and care recommendations to help primary care facilities improve their structural model to help manage and control the pandemic and to respond to the health needs of their patients.^18^ The design of these recommendations incorporated guaranteed safe access to health‐care centres, gradual face‐to‐face care for chronic patients, and proposals for the care of institutionalized patients.^18^

Locally, efforts to minimize the risk of infection due to the COVID‐19 pandemic have interrupted the care and follow‐up of chronic patients, as well as scheduled diagnostic tests. Furthermore, advances made to date for more PCC in both national and regional health‐care services are converting patients and their caregivers in the principal responsibility of their self‐care.^19^ The effects of the COVID‐19 era require rethinking the current health‐care system structure and evaluating the stability of previous advances made in medical care. The lack of stable and significant advances emphasizes the need for more practical guidance for PCC implementation and for specific criteria and indicators to cover all dimensions of PCC.^20^ It is important to preserve the care of the patient, to integrate their experiences when processes are redefined and to maintain their participation in decision‐making processes.^20^, ^21^

This study aimed to identify the priorities of PCC and to establish criteria and indicators with which health services can use to model stable and reliable PCC goals and objectives in both the current and post‐COVID‐19 era.

## 2 METHODS

**FIGURE 1:**
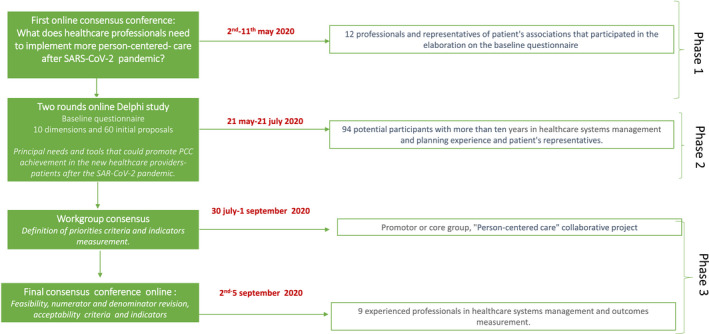
Flowchart of study phases: consensus conference, Delphi study and final workgroup consensus conference

A qualitative study was conducted between May and July of 2020. This study was performed in three phases (Figure 1). In the first phase, a consensus meeting was held to identify health‐care professionals' needs in reaching the PCC target after the pandemic caused by severe acute respiratory syndrome coronavirus 2 (SARS‐CoV‐2). In the second phase, the Delphi methodology^22^ was used to identify the priorities of PCC in the time of COVID‐19. Meanwhile, a consensus conference established indicators and criteria for achieving PCC goals. This study was approved by the project's evaluator commission of the local university (reference number DEE.IMS.02.20/04.19).

Development of a baseline questionnaire was initiated by applying the Delphi technique to an online consensus meeting. For the Delphi study, an online platform made it possible to manage invitations and reminder messages, respond confidentially, and analyse the received responses. Google Hangouts was used for the online consensus meetings.

### 2.1 Participants

The first work group participated in the development of the baseline questionnaire and included 12 professionals and representatives from patient associations.

For the Delphi study, 94 potential participants were identified from various health services through informal contact and the snowball sampling technique. The inclusion criteria were as follows: at least 10 years of experience as a health‐care professional (doctors and nurses), health‐care institutions and department managers, information systems experts, health quality experts, public health experts, academics and patient representatives. Refusal to participate was the exclusion criterion. Voluntary participation began after receiving information about the study objectives and the methodology to be applied. The first email was sent to inform the participants about the study objectives and scope, the confidentiality of the responses, and included a participation agreement form. After receiving the signed participation agreement, a second email was sent with methodological instructions and a link to the online response register.

In the final consensus meeting, another nine experienced health‐care system management and evaluation professionals established the PCC criteria and indicators. They reviewed their relevance and acceptability, as well as the indicator definitions, the viability of the calculation formula and potential information sources. These professionals adapted the criteria to formulate the indicators using corresponding numerators and denominators. In this phase, there was no patient participation.

In this study, there was a promoter group, or core group, which included eight professional PCC experts. Their role was to synthesize the proposals in the different phases of the study. (see Table 1).

**TABLE 1:** Profile of workgroup participants

Working group	Gender	Position and experience
Core Group (*n* = 8)	75% males y 25% females	50% physicians, 50% other (psychologists, sociologists and nurses), >25 years of experience in management (including patients associations), health policy or academic positions
First online consensus conference (*n* = 12)	50% males y 50% females	66% physicians, 17% nurses, 17% other, >20 years of experience in management positions (including patients associations), health policies, nursing, training or public health
Delphi study (*n* = 94)	30% males y 70% females	40% physicians, 50% nurses, 10% other, >15 years of experience in management and leadership positions (academics, nursing, management, family medicine, public health, technical staff and representatives of patient associations)
Final consensus meeting (*n* = 9)	100% females	All physicians, >10 years of experience as technical staff in information systems and quality measurement

### 2.2 First consensus meeting

The first consensus meeting was conducted online and consisted of a brainstorming session in which professionals and patients were asked, ‘What do healthcare professionals need to implement more person‐centred care during and after the SARS‐CoV‐2 pandemic’? These online consensuses determined the first pool of priorities related to professionals' needs in achieving PCC after the pandemic. Each professional or patient provided a group of proposals from different dimensions using a common approach. The first selection round included all the suggested proposals.

### 2.3 Delphi study

The suggested proposals for PCC priorities were rated using a 0‐to‐10‐point scale; proposals rated between 8 and 10 points were considered absolute priorities, proposals rated between 5 and 7 points as medium priorities, and proposals rated between 0 and 4 points as relative priorities. The following aspects determined the acceptance grade of agreement: average proposal score (between 7 and 8 points) and the coefficient variability (higher variability; more significant homogeneity within scores). This method achieved sufficient consensus that allowed for the definitive acceptance or exclusion of the proposal when the average was below or above these scores.

The second round worked to improve the wording of the first‐round items and to clarify their meaning and allowed for the inclusion of new proposals. The same rating scale was applied in this round. Nine or ten points indicated a high‐grade consensus (achieved in 50% of the proposals); scores between 49% and 40% indicated moderate consensus; and the rest were eliminated. The participants' average scores from the first round were made available for review in the second round.

### 2.4 Consensus meeting

From the list of identified PCC priorities, the core group established by consensus the criteria and indicators to measure the levels of achievement and defined the numerators, denominators, reference standards, data sources and periodicity. Nine experienced management and outcome‐measurement professionals provided opinions to help with the initial reformulation of the proposals in the following aspects: indicator's feasibility, modification of the numerator's calculation, denominator and the establishment of acceptability. This phase allowed for the achievement of a higher consensus on the proposed metrics of the results from the previous phase.

## 3 RESULTS

The first panel of experts suggested a group of 125 proposals for priorities categorized among 11 dimensions. The core group analysed these proposals based on productivity terms: the number and consistency of ideas presented by each expert and ideas that were repeated among different panels of experts. The analysis permitted the elimination of duplicate ideas, the reformulation of selected proposals using the Delphi technique and for the distribution of proposals into the 11 dimensions. After reaching a consensus among the first group of experts, the evaluation of the initial proposals ultimately resulted in 10 dimensions and 60 proposals. A revision of the text for increased comprehension contributed to the improvement in the wording of 11 proposals. These revised proposals constituted the baseline questionnaire used in the Delphi technique. The dimensions included in the baseline questionnaire were described as follows:

1. Leave no one behind or care for the most vulnerable people: During the first wave of the pandemic, the suspension of assistance activities had an ironic effect, causing the most damage to those with a higher level of vulnerability.
2. Safe use of health‐care resources: Create safe centres and spaces with reduced risk of contagion.
3. Health literacy: Provide more information to citizens to make them more capable of making and increasing responsibility for their own health‐care decisions.
4. Self‐care and patient autonomy: Provide information for the promotion of self‐care for the patient's benefit and for the interest of the community.
5. Persistence and adherence to pharmaceutical and non‐pharmaceutical treatments: Promote higher treatment adherence and encourage patients to adopt healthy habits for improved health status.
6. Adequacy in health care and social resources: Efficient use of health resources for equal access and adaptable conditions for vulnerable groups.
7. Pursuit of increased efficiency: The incorporation of information derived from telemedicine interventions in to the medical records to guarantee patient safety and reduce unnecessary visits.
8. Improve the patient experience: Pursue a positive patient experience with health‐care services facilitate interaction between families and patients during quarantine using communication technologies and strengthening the humanization of care in the last stages of end‐of‐life.
9. Health sector recovery through community network: Collaboration between the community and social services to explore new ways to identify patients' actual needs that have emerged from this pandemic.
10. Care of health‐care workers (linked to the Quadruple Aim model)**:** Promote the well‐being of and provide emotional support for health‐care professionals to allow them to continue caring for others.

The first round had a high participation rate (81%) with a panel of 76 professionals; the second round included 60 experts (63.8% participation rate). From the first round, 32 priorities were withheld, another nine proposals were rejected, and eight new ones were added. The rejected proposals from the first round are listed in the Supplementary Material.

At the end of the second round, 28 priorities reached a high consensus (see Table 2). None of the proposals included in the health‐literacy dimension had a sufficient level of consensus for inclusion; 10 had a moderate consensus level, and 21 were rejected (Table 3). The core group evaluated these 28 priorities, which were then used to formulate the corresponding criteria and indicators for monitoring the level of success achieved in each dimension.

**TABLE 2:** Delphi study results. Priorities in achieving person‐centred care in the COVID‐19 era

	*N*	Mean	SD	CV	% ≥9	Round^a^
01. Leave no one behind, care for more vulnerable people						
Promote the communication and the coordination between hospital and primary care to create and impulse shared clinical and management structures	76	9.1	1.2	0.1	76.3	1
02. Safety in the use of health‐care resources						
To involve the patient in making decision on his health among all health‐care system levels and social institutions for the elderly and people with functional diversity	76	8.9	1.2	0.1	67.1	1
Reduce the possibility of contagion in the access area to health‐care centres by differencing the workflows to patients based on the suspicion of COVID‐19 and providing protection elements to patients who don't carry it on^b^	60	8.8	0.9	0.1	65.0	2
To assess health determinants by nursing professionals, at primary and hospital care levels, in chronic patients and in their family setting	60	8.4	1.6	0.2	53.3	2
To promote the preventive medicine in the primary care by improving the prevention and information on patient health.	74	8.4	1.4	0.2	52.7	1
03. Health Literacy						
04. Self‐care and patient autonomy						
To promote the individual responsibility on self‐care and its benefits on collective health	75	8.6	1.3	0.2	52.0	1
05. Persistence and adherence to pharmaceutical and non‐pharmaceutical treatment						
To promote shared decision making about treatment by implementing health goals to favour non‐pharmaceutical interventions to common problems and avoid medicalization	74	8.5	1.6	0.2	59.5	1
To promote the message that the pharmaceutical treatment is not the unique therapy and decision related to patients eating and exercise habits, toxic habits and other habits prevent contagion	73	8.4	1.8	0.2	57.5	1
To motivate patients to achieve healthier habits, attitudes and skills necessary to maintain a healthy lifestyle, to propose changes in the environment that facilitate healthy living conditions and reinforce positive health	60	8.0	2.2	0.3	50.0	2
06. Adequacy in health care and social resources						
To promote social well‐being and care in more vulnerable groups (elderly, migrants, dependent people or social exclusion risk, home care) providing a health budget adapted to their needs	73	8.5	1.5	0.2	58.9	1
To promote the nurse case manager role, to help and provide information about patients and their caregivers in order to facilitate an integrated care, being the reference for most vulnerable patients under her/his charge, carrying integrated care, affording advice during hospital stay, and discharge, checking the patient's conditions, if family support is available or no, and when social support is absent, search and manage an admission to social institutions	60	8.1	2.4	0.3	55.0	2
To establish alert criteria in social‐health centres that activate immediate monitoring actions adapted to the attended population	73	8.5	1.5	0.2	54.8	1
To predict the availability of multidisciplinary ‘intervention teams’ to the possibility of overload in some centres or within community groups plus to its management difficulty (social‐health centres, institutions for functional diversity)	73	8.4	1.6	0.2	54.8	1
To update the ‘resource maps’ of all administration's health care and social services (National, regional and municipal) including human, technological and material resources (Beds or vacancy in social‐health or conventional centres)	73	8.4	1.5	0.2	53.4	1
To enable multifunctional areas for rapid display and adaptation when ICU overload happen^b^	60	8.2	2.1	0.3	51.7	2
07. Search for higher efficiency						
To offer integrated care by facilitating PC professionals’ access to hospital care information (Digital health record, imaging resources, etc)	73	9.2	1.5	0.2	80.8	1
To promote and to impulse ‘Do‐Not‐Do’ recommendation as care goals avoiding unnecessary procedures and tests of questionable utility	73	9.0	1.5	0.2	78.1	1
To build multidisciplinary groups based on hospital, familiar and social approaches in order to address a problem from an integrative vision and adapted to the urgency degree and the information should be available in each level, institution and managers	60	8.1	2.4	0.3	56.7	2
To offer an integrated care facilitating for the emergency and out‐hospital services the access to hospital care and primary care information (Electronic Health Records, imaging resources, etc). To integrate in the Electronic Health Records information on the care provided by these services in order to make it accessible to PC and Hospital care professionals	60	8.2	2.2	0.3	55.0	2
To avoid the patient referral cancellation and substitution in hospital care by telemedicine assistance using image sending (photographs, echography and reports) to support diagnosis in PC or other levels in order to guarantee efficacy and patient safety	60	8.2	2.1	0.3	51.6	2
08. To improve patient experience						
To use technologies that facilitate communication between isolated patients and their families (Tablet, telephones etc)	74	8.4	1.7	0.2	64.9	1
To update protocols of accompaniment in hospital stay and during palliative care (last moments of life) in order to promote humanity	74	8.5	1.7	0.2	62.2	1
To guarantee after the outbreak the early restoration of personalized assistance (nurse and doctors) as a key aspect of primary care quality^b^	74	8.4	1.9	0.2	60.8	1
09. Health sector recovery through community network						
To increase awareness about a responsible use of health care and the use of protection elements, as the use of new technologies and available digital channels to monitor health status in case of isolation	74	8.4	1.5	0.2	54.1	1
The cooperation between PC and social services through a common ‘Centre Plan’ in order to detect the person's needs, identifying the community most prevalent health problems, as the social or environmental determinants and establishing strategies to resolve them	60	8.2	1.9	0.2	51.7	2
10. Take care of health‐care workers (Quadruple milestone)						
To facilitate in the centre or telematic psychological assistance service for the professionals	72	8.5	1.5	0.2	66.7	1
To facilitate self‐care guidelines for the professionals (protection and prevention elements, stress management etc)	73	8.3	1.6	0.2	58.9	1
To incorporate the update of knowledge about distress prevention in the compulsory long‐life learning for health professionals	72	8.4	1.6	0.2	52.8	1

Abbreviations: CV, coefficient of variation (range 0 a 1); SD, standard deviation; % ≥9, Percentage of participants who rated the proposal with 9 or more.

^a^ In which round a sufficient consensus was reached for the proposal.

^b^ Implemented in a wide scope at the end of this study.

**TABLE 3:** Proposals with moderate consensus levels and rejected proposals with insufficient consensus levels

	*N*	Mean	SD	CV	% ≥9	Round^a^
01. Leave no one behind, care for more vulnerable people						
To coordinate with social services the identification on patients with no technological resource and facilitate to them devices and training for telemedicine	60	7.6	1.6	0.2	30.0	2
To create primary care and hospital care units in residential homes to ensure a safe and effective care and to increase the kindness in patient experience	60	7.7	1.7	0.2	26.6	2
To create a community agent figure to facilitate information and ensure follow‐up continuity for elderly, families and personas without previous knowledge of new technologies	60	7.2	1.3	0.2	15.0	2
To coordinate both socioeconomic and health interventions. To ensure a minimum vital income to persons with unstable work or affected by submerged economy forced to lockdown or isolation	60	7.5	2.4	0.3	35.0	2
02. Safety in the use of health‐care resources						
To guarantee a safe identification of COVID‐19 patients assisted within telephonic consultation	60	8.5	1.1	0.1	45.0	2
03. Health Literacy						
To train medical and nurse staff in proximity digital communication using the image of reference staff, offering people preventive and educational information	60	7.3	1.9	0.3	16.7	2
To offer from the patient's school courses conducted virtually by primary care services to educate in self‐care, facilitating general and specific educational material	60	7.7	1.8	0.2	21.7	2
To guarantee quality and safety in remote information: updated official information offered via telephone and virtual calls and available on the website	60	7.9	1.7	0.2	36.6	2
Health education to empower patients in healthy habits that contribute and maintain a good immunity system, and essential aspects to avoid contagion, at primary and hospital healthcare (hands washing, mask use, home ventilation, social distance and vaccination recommendations)	60	8.2	1.7	0.2	48.4	2
To include in virtual personal health record or app, vaccination calendar and institutional information (available on official website) with pop‐up reminders for patients and caregivers	60	7.6	1.8	0.2	25.0	2
04. Self‐care and patient autonomy						
To facilitate access to virtual personal health record or by official health‐care system apps to educate and aware chronic patient on health status and to activate in self‐care	60	8.1	1.6	0.2	41.6	2
To activate youth and young adults in healthy habits via health apps of local health‐care council/ health‐care department/health‐care area and to guide elderly in maintaining vital functions (mobility) through simple workshops	60	7.7	1.6	0.2	25.0	2
To elaborate and to facilitate a checklist about COVID‐19 protection elements to apply in the family or personal circles at home and in daily activities: way to work, to the supermarket or stores, during sport activities etc	60	7.7	1.4	0.2	21.6	2
05. Persistence and adherence to pharmaceutical and non‐pharmaceutical treatment						
To include in the health‐care department/area app instruction for medication safe use, reminders and motivation messages with active follow‐up for patients with special needs and/or for their caregivers	60	7.6	2	0.3	26.7	2
To involve community pharmaceutics and social services with PC teams to define reinforcement therapeutic adherence strategies and to avoid medication administration errors in the elderly	60	7.9	2.2	0.3	45.0	2
To include in the long‐life learning for health professionals’ courses about rational use of medication	60	8.1	2.1	0.3	48.3	2
06. Adequacy in health care and social resources						
To establish an emergency psychological care for future situations similar to the experienced during the COVID‐19 pandemic, and make it available by out‐hospital medical emergency services and via medical telephone coordination centres for unbalanced psychiatric patients in similar situations (2 months lockdown) that need long time care from medical emergency services	60	7.5	2.2	0.3	31.7	2
07. Search for higher efficiency						
08. To improve patient experience						
To include auditor patient figure to incorporate efficiently perception about accessibility, care continuity and patient safety	60	6.4	2.1	0.3	13.3	2
To offer the person perception of cohesion between services for coordinated response in the disease and follow‐up between PC and hospital teams	60	7.1	2.3	0.3	18.4	2
To provide the person a virtual platform to register the need of emotional or health‐care support	60	7.1	2.1	0.3	16.7	2
To clarify and update protocols about access to health‐care centres and support for vulnerable patients, in pregnancy, children and palliative care patients etc	60	7.8	2.6	0.3	48.3	2
To train patients in self‐care during home isolation, and health education for the patients and their social and family environment in pandemic phases such as COVID‐19	60	7.9	2.3	0.3	48.3	2
09. Health sector recovery through community network						
To maintain the contact between the patients' associations, the citizens, NGOs and the health‐care centre to promote preventive measures and social support in the neighbourhoods	60	7.7	2.1	0.3	26.7	2
To cooperate actively from the PC centres, the public health staff, education centres in the programs and campaigns offering virtual and face‐to‐face information towards young people about the zoonosis, the importance of individual and collective health education, and how it affects the ecosystem	60	7.2	2.0	0.3	23.3	2
To promote in the cities a sustainable mobility avoiding the worsening of pollution and therefore the pandemics, and likewise promoting healthy lifestyle and physical activity	60	7.2	2.4	0.3	31.6	2
To maintain an active contact of health‐care services with the local councils, so that they take responsibility knowing the situation of most vulnerable people by supporting and collaborating decidedly with municipal health structures	60	7.7	2.2	0.3	43.3	2
Health assets mapping during the pandemic, de‐escalation, and the 'new' normality	60	7.9	1.9	0.2	38.3	2
10. Take care of health‐care workers (Quadruple milestone)						
To create ‘a wellbeing room’ in hospital facilities for health‐care works and spaces to relax	60	7.6	1.9	0.2	31.7	2
To promote the occupational medicine and nursing services and the occupational risk prevention services that permit an active surveillance of professional's health	60	8.2	1.8	0.2	48.4	2
To support family reconciliation, it was an added stress factor during the pandemic due to the lack of relatives and schools’ support	60	7.6	2.2	0.3	33.4	2
To incorporate to the curricula and long‐life learning system updated information about preventive actions and measures to stress coping	60	8	1.9	0.2	48.4	2

Abbreviations: CV, coefficient of variation (range 0 a 1); SD, standard deviation; % ≥9, Percentage of participants who rated the proposal with 9 or more.

^a^ In which round a sufficient consensus was reached for the proposal.

The reformulation criteria and indicators were possible in 20 of the 28 identified priorities, including the construction of their corresponding indicators. The eliminated priorities have already been systematically used in the health‐care system.

Based on these selections, 20 criteria and 21 indicators were established to monitor the implementation of PCC and the levels of achievement (Table 4). Among these criteria, three were not included, considering that they are currently applied systematically in health‐care centres. For further details, see Supplementary Material 2.

**TABLE 4:** Prioritized proposal criteria and indicators for implementation and monitoring of levels of achievement in this Delphi study

Criteria	Indicator formula
Proposals for the Electronic Health Records (EHR)	
*EHR_1* Reorienting the focus of the electronic digital record towards person‐centred care (PCC) permit the usage of clinical parameters and diagnosis for a global vision of the person status (psychosocial aspects: occupational (active, retired, unemployed, and work sector), capacity for self‐care, and problems and risks that imped to enjoy activities, have a restful sleep, a good appetite etc)	Numerator: number of patients with psychosocial, occupational aspect information and self‐care capacity evaluation registered in a unique Electronic Health Records Denominator: total number of patients attended at the health‐care centre
*EHR_2* Adaptation of Electronic Health Records for a personalized follow‐up using telemedicine programs. To incorporate shared virtual communication channels permitting online consultations, resolving doubts of patients and caregivers	Numerator: number of patients using virtual commination channels with professionals during 3 months. (online consultations and usage of virtual personal health record) Denominator: total number of patients attended at the health‐care centre
*EHR_3* Integrate social and clinical registers. To permit the interoperability and accessibility to clinical register information of local, regional and national health care and social wellbeing services (IMSERSO, social wellbeing system)	Numerator: number of dependent patients (60 or higher points in Barthel index) with social and clinical information incorporated in the unique Electronic Health Records Denominator: total number of dependent patients of the health area
*EHR_4* Inter‐consultations, referral and support in making decisions using mHealth solutions	Numerator: number of digital inter‐consultations and referrals processed by specialties services Denominator: total number of inter‐consultations and referrals processed in the health area
*EHR_5* Incorporating operative alerts system for the benefit of patient's safety	Numerator: number of 'Not‐to‐Do' practices associated with operative alerts for clinical decisions incorporated in the commitment to quality Denominator: total number of 'Not‐to‐Do' incorporated in the commitment to quality
*EHR_6* Access to patient's occupational register (temporary incapacity for work) to evaluate the work conditions and determine its effects on disease progress	Numerator: number of workdays lost due to pending diagnosis test or referral to different medical specialty service Denominator: number of patients with work activity attended in the health for a temporary incapacity for work
*EHR_7* To incorporate social prescription in Electronic Health Records (To permit linking non‐medical social networks from the community to the primary care system. These networks or sources could include physical activities, learning activities, volunteerism, mutual assistance, fraternity and self‐help groups, creative and arts lessons, as legal guide and support for parental problems etc)	Numerator: number of patients with chronic conditions participating (for 3 months) in activities with social prescription approach Denominator: total number of patients with chronic conditions attended in the health area during the last year
Organizational/procedures	
*OP_1* To ensure availability of alternatives for people with digital gap to avoid its impact on assistance quality	Numerator: number of patients not included in telemedicine programs (expect those that rejected inclusion due to digital analphabetism or other reasons) Denominator: number of patients included in telemedicine programs or use mHealth solutions supervised by health area professionals
*OP_2* Access to updated maps of social and health resources, specially to address the situations of most vulnerable persons	Numerator: number of professional of the health area that accessed or downloaded from the intranet information about social and health resources during the last 6 months Denominator: number of active professionals in the health area during the last 6 months
*OP_ 3* Availability of multidisciplinary intervention teams (professional teams of different disciplines that act in unexpected situations/situation of force) qualified to assist special collectives or centres in overflowed services	Numerator: number of professionals involved in multidisciplinary seminaries/workshops/work m to address overflowed situations (health crisis) Denominator: number of active professionals in centres of the health area
*OP_4* To extend equally the use of high‐resolution consultation to polymedicated patients as in the case of those with preferential access to consultations and diagnosis tests (preferential health‐card access) applying producers to ensure its correct usage	Numerator: number of polymedicated chronic patients included in high‐resolution consultations procedures Denominator: number of polymedicated patients (with more than 5 drugs per day) attended during the last three months Numerator: number of patients with preferential access attended in less than 10 minutes respect the scheduled time Denominator: number of patients with preferential access attended in the last three months in the centre
*OP_5* To measure persons experience systematically by social and health‐care systems, linking these results to management agreements	Numerator: number of patients with positive experience with the organization and the received assistance (score higher than percentile 75 of the applied scale) Denominator: number of patients that participate in patient experience analysis studies
Activation of persons as health asset agent	
*ACTV_1* Patients with personalized care plan, including lifestyle information (eating habit, physical activity and sleep), impact on social and occupational activity, pharmaceutical and non‐pharmaceutical treatment and comorbidities management	Numerator: number of patients suffering of chronic conditions with personalized care plan established with health‐care agents Denominator: number of patients suffering of chronic condition (3 or more) attended in the health‐care centre
*ACTV_2* Persons with adequate adherence with the agreed therapeutic goals, for pharmaceutical and non‐pharmaceutical treatment (For example, INR therapeutic goals range percentage, daily distance or steps)	Numerator: number of patients with adequate adherence to IAP Denominator: number of patients with IAP
*ACTV_3* Complex chronic patients (2 + 3) with the support and intervention of nurse cases manager to increase the high‐resolution consultations, to ensure coordination between specialties and another assistance levels, including psychosocial care and reducing safety incidents during referrals or transitions	Numerator: number of complex chronic patients included and activated in nurse cases manager list Denominator: number of complex chronic patients (2 + 3) attended in the centre
New goals	
*GOAL_1* To review procedures and resources to consider relevant relational aspects for isolated patients and their families	Numerator: number of isolated patients/families with positive experience with the organization and the received assistance (score equal or higher than percentile 75 of the applied scale) Denominator: number of isolated patients /families that participated in patient experience analysis studies conducted by the health area
*GOAL_2* To address the low‐value practice (Not‐to‐Do) that impact negatively on patients and the sustainability of public health system	Numerator: number of management agreements related to low‐value practices Denominator: number of management agreements objectives addressed by health‐care services and assistance units
*GOAL_3* To increase care quality establishing the quadruple approach as target	Numerator: number of professionals that valued the organization positively, leadership styles and work wellbeing (score equal or higher than 75 percentile of the applied scale) Denominator: number of professional in the health area
*GOAL_4* To improve person's life conditions. To reduce days of work leaves related to health condition (For example to perform a test or for medical consultations)	Numerator: number of patients requiring an accreditive document of diagnostic tests and consultations attendance to justify absent from work Denominator: number of patients that attended diagnostic tests or assisted in the centre
*GOAL_5* To Incorporate STOPP‐START criteria or potential inadequate drugs list in pharmacy services to avoid safety incidents with medication of persons assisted in social‐health centres	Numerator: number of prescriptions for patients over 65 years with STOP‐START criteria/ list of potential inadequate drugs Denominator: total number of prescriptions for patients over 65 years attended at the centre

In the consultation round, the 20 criteria were included in the following four dimensions: proposals for electronic health records, organization and procedures, patient participation and new goals. For each of these 20 criteria, clarifying information was added and 12 were revised for further improvement. In addition, the information sources and reference standards were changed in other four criteria, and redundant information was deleted from one criterion.

## 4 DISCUSSION

The COVID‐19 pandemic has significantly modified the priorities for the achievement of PCC in the Spanish health system, specifically access to primary care while preserving patient and community safety. Public and scientific institutions have proposed a new organizational model to restore public health services beyond the control of COVID‐19.^18^, ^19^ However, if there are no metrics or measurement criteria, these initiatives will remain as good intentions. These results showed proposals for PCC in nine work dimensions and 28 identified priorities that would improve the capabilities of the health‐care system to improve health outcomes while respecting patient values. In addition, these proposals support the quadruple aim approach, which considers the need for care for health‐care professionals, without whom it is impossible to achieve a better quality of care.^23^, ^24^, ^25^ Lastly, these proposals are in accordance with the value‐based health‐care approach.^1^, ^2^

The pandemic caused by SARS‐CoV‐2 has had a devastating impact on Spain.^26^ The incidence of cases, the burden of patient care, the number of deceased patients and the number of infected (and also deceased) professionals were all higher in Spain than in neighbouring and less developed countries.^15^, ^27^ In this health crisis, the quality of the health‐care system has now been questioned and positive opinions of it have diminished.^28^, ^29^, ^30^ It is predicted that there will be an increased demand for the review of current approaches, procedures and goals in the coming months.^31^ The path undertaken to implement PCC should not be obstructed, and it may be necessary to re‐launch the interventions aimed at this objective throughout the health system. Measuring the degree of achievement of the relevant objectives is one way of contributing to this objective.

On an institutional basis, the first step towards post‐pandemic recovery was the National Commission's decision for social and economic reconstruction (the so‐called Cajal project, which is in progress). Among the recommendations approved in a plenary session by the Spanish Congress of Deputies were the need to strengthen support for professionals, the aim to achieve a higher level of patient participation in making decisions, and the promotion of patient wellbeing, safety and autonomy. It was also approved to promote the digitalization and permanent incorporation of primary care medical records in primary care centres, to use telemedicine and to provide telephonic assistance.^17^, ^21^, ^32^ These goals are included among the priorities of this study.

To our knowledge, this is the first study to analyse the priorities necessary to achieve PCC implementation goals in the COVID‐19 era. Scientific societies, such as the Spanish Society of Public Health and Sanitary Administration^33^ (*Sociedad Española de Salud Pública y Administración Sanitaria*; SESPAS) and the Spanish Society of Health Quality^34^ (*Sociedad Española de Calidad Asistencial*; SECA), presented a set of recommendations for health‐care system recovery after the impact of the pandemic on how to respond in the case of a pandemic resurgence. In neighbouring countries, similar criteria, such as the Vocal Project in the British National Health Service, have had a positive impact on the health‐care system during the pandemic. They led to the increasing use of telemedicine in local and national health‐care centres,^35^ structural and operational revisions of patient‐centred ambulatory care and pharmacy services,^36^ and the organized approach to managing loneliness and social isolation via a public campaign during the lockdown and the social‐distancing period of the COVID‐19 pandemic.^37^

These proposals could be utilized by managers and health authorities to assess the viability and adaptation of the policies that are currently in practice. A shared effort from all stakeholders, such as policymakers, patient representatives, physicians and clinical staff, and public health researchers, is necessary to move these ideas from proposals to actions in the development of the PCC approach. Focusing on the operative adjustments to electronic health records, self‐care promotion, procedural redesign and the wellbeing of health‐care professionals will serve as tools in the achievement of better health‐care outcomes.

### 4.1 Limitations

No analysis was conducted as to how these recommendations can be put into practice. This study considered the professionals’ perspectives, and not just the organizational and management experience, which usually do not represent the clinician's or other health‐care professional's point of view. Due to the particularity of the differences between the territorial health services, the information collected and used to calculate some of the proposed indicators may require structural modifications.

This study was conducted in Spain in the context of a national health model. Although the pandemic's overall impact on health systems may have varying intensities, the changes made in response to its effects have been similar. In this sense, although the data should not be extrapolated to other health models, the results can offer clues on how to proceed in the short and medium terms.

In this study, the feasibility of these proposals served to evaluate their priority. The proposals are accompanied by criteria that indicate the direction of progress and indicators to determine if the progress is moving in the correct direction. This study is moving in this direction.

The changes to be implemented should be accepted by the workgroups (those responsible for putting them into practice) and accompanied by tools and metrics that can monitor the levels of achievement. Otherwise, these proposals will be viewed solely as good intentions.

## CONFLICT OF INTEREST

This study has been funded by Merck, Sharp and Dohme Research Laboratories, Spain. Design, analysis and interpretation of data were conducted exclusively by the authors.

## AUTHOR’S CONTRIBUTION

All authors participated in the conception and design of this study. Mira JJ, Blanco M, Cheikh‐Moussa K, Solas O, Alonso A and Guilabert M collaborated in the processing and analysis of the data. Mira JJ, Cheikh‐Moussa K, Solas O, Alonso A and Guilabert M participated in the interpretation of the results. Mira JJ, Cheikh‐Moussa K and Guilabert M collaborated by writing the draft of this manuscript. All authors performed a critical review of the content of the manuscript. All authors have approved the final version that is sent to the journal.

## Supporting information

Supplementary Material

## Data Availability

The data that support the findings of this study are available from the corresponding author upon reasonable request.
