# Supplementary material for: Proposals for person‐centred care in the COVID‐19 era. Delphi study
Source: Health Expect. 2021 Feb 27;24(2):687–99. doi: 10.1111/hex.13218 (PMC8013374; doi:10.1111/hex.13218)
Supplement: Supplementary file 1 — Supplementary Material [file HEX-24-687-s001.docx]

**Supplementary material 1. Withdrawal proposals in the first round.**

| **01. Leave no one behind, care for more vulnerable people** | N | Mean | S.D. | CV | % >= 9 | Round^ |
| --- | --- | --- | --- | --- | --- | --- |
| To establish cooperation agreement between heath care, education and business sectors to promote apps and telemedicine systems adapted for the current needs. | 76 | 7.0 | 1. | 0.2 | 18.4% | 1 |
| To create management units of mobile health care assistance for home-monitoring of vulnerable persons with special situation. | 76 | 6.8 | 2.5 | 0.4 | 23.7% | 1 |
| **02. Safety in the use of healthcare resources** | N | Mean | S.D. | CV | % >= 9 | Round^ |
| To establish permanently access control at health care centres entrance facilitating rapid diagnostic equipment (Infrared thermometer, and rapid detection test) plus to COVID-19 symptoms questionnaire. | 76 | 7.6 | 2.1 | 0.3 | 39.5% | 1 |
| **03. Health Literacy** | N | Mean | S.D. | CV | % >= 9 | Roun^* |
| To develop in the most used social media profiles for the PC centres (Facebook, twitter y Instagram) to facilitate health information, recommendations and educational lessons about selfcare of the chronic patient. | 76 | 6.6 | 2.2 | 0.3 | 15.4% | 1 |
| To inform about the importance of emotional status for the prevention of CVID-19 contagion, and to maintain an adequate health status. | 74 | 7.5 | 1.5 | 0.2 | 23.0% | 1 |
| To integrate in schools and high schools the school nurse figure in order to facilitate information about infectious diseases, protection measures and vaccination. | 75 | 5.7 | 2.9 | 0.5 | 13.3% | 1 |
| **04. Self-care and patient autonomy** | N | Mean | S.D. | CV | % >= 9 | Round^ |
| To promote apps with passive voice elements, watches with pulse sensors, to active patients and/or their caregivers in their selfcare. | 75 | 7.1 | 1.7 | 0.2 | 21.3% | 1 |
| To facilitate COVID-19 detection programs and risk level checklist to patients through health care department website, adapted to age groups, in order to evaluate their risk level. | 75 | 7.1 | 1.4 | 0.2 | 14.7% | 1 |
| **05. Persistence and adherence to pharmaceutical and non-pharmaceutical treatment** |  |  |  |  |  |  |
| **06. Adequacy in healthcare and social resources** |  |  |  |  |  |  |
| **07. Search for higher efficiency** |  |  |  |  |  |  |
| **08. To improve patient experience** | N | Mean | S.D. | CV | % >= 9 | Round^ |
| To provide PC centres a community agents network to establish telephonic connections with patients highlighting on their needs through a casual conversation. | 73 | 6.6 | 1.8 | 0.3 | 13.7% | 1 |
| **09. Health sector recovery through community network** |  |  |  |  |  |  |
| **10. Take care of health care workers (Quadruple milestone)** |  |  |  |  |  |  |
| ^ In which round a sufficient consensus was reached for the proposal rejection. |  |  |  |  |  |  |
| S.D. standard deviation; CV coefficient of variation (range 0 a 1); % >= 9 Percentage of participants who rated the proposal with 9 or more. | | | | | | |

**Supplementary material 2. Criteria definition, indicator formula and information source for PCC achievement.**

| **Proposals for the Electronic Health Record (EHR).** | | | | | |
| --- | --- | --- | --- | --- | --- |
| **Electronic Health Record (EHR).** | |  | | |  |
| **CODE** | **CRITERIA DEFINITION** | **INDICATOR FORMULA** | | | **SOURCE AND PERIODICITY** |
| **EHR_1** | Reorienting the focus of the medical record toward person-centred care (PCC) permits the usage of clinical parameters and diagnosis for a global vision of the person status (psychosocial aspects: occupational (active, retired, unemployed, work sector), capacity for selfcare, and problems and risks that imped to enjoy activities, have a restful sleep, adequate eating habits, etc.) | **Numerator:** number of patients with psychosocial, genogram, occupational information and self-care capacity evaluation registered in a unique digital medical record.  **Denominator:** total number of patients attended at the healthcare centre (hospital or primary care).  **Standard reference:** >25% in the first year, >35% in the second, >40% in the third year, >45% from fourth year. | | | **Data source:** medical record  **Periodicity:** information recovery will be by semester the first two years, after this period with be quarterly. |
| **EHR_2** | Adaptation of medical record for a personalized follow-up using telemedicine programs. To incorporate shared virtual communication channels permitting online consultations, resolving doubts of patients and caregivers. | **Numerator:** number of patients using virtual commination channels with professionals during 3 months. (online consultations and usage of virtual personal medical record).  **Denominator:** total number of patients attended at the health care centre.  **Standard reference:** >25% in the first year, >45% in the second, >55% in the third year, >65% from fourth year. | | | **Data source:** appointment with a health care professional. |
| **EHR _3** | Integrative social and medical record. To permit the interoperability and accessibility to clinical information of local, regional and national health care and social wellbeing services (IMSERSO, Social Insurance system). | **Numerator:** number of dependent patients (60 or higher points in Barthel index) with social and clinical information incorporated in the medical record  **Denominator:** total number of dependent patients of the health area.  **Standard reference:** >35% in the first year, >55% in the second, >70% in the third year, >95% from fourth year. | | | **Data source:** medical record  **Periodicity:** quarterly. |
| **EHR _4** | Inter-consultations, referral and support in making decisions using applications available in patients' virtual medical record. | **Numerator**: number of digital inter-consultations and referrals processed by specialties services.  **Denominator**: total number of inter-consultations and referrals processed in the health area.  **Standard reference:** >75% in the first year, >95% from the second year. | | | **Data source:** unique digital clinical record  **Periodicity:** quarterly. |
| **EHR _5** | Incorporating operative alerts system for the benefit of patient’s safety. | **Numerator:** number of 'Not-to-Do' practices associated to operative alerts for clinical decisions incorporated in the commitment to quality.  **Denominator**: total number of 'Not-to-Do' incorporated in the commitment to quality  **Standard reference:** >10% in the first year, >20% in the second, >35% from the third year. | | | **Data source:** Autonomous community health care authorities report (management agreement, corporative document, technological evaluation agency, quality department).  **Periodicity:** annual. |
| **EHR _6** | Access to patient's occupational register (temporary incapacity for work) to evaluate the work conditions and determine its effects on disease progress. | **Numerator:** number of work days lost due to pending diagnosis test or referral to different medical specialty service.  **Denominator**: number of patients with work activity attended in the health for a temporary incapacity for work.  **Standard reference:** >25% in the first year, >35% in the second, >45% in the third year, >55% from the fourth year. | | | **Data source:** medical record.  **Periodicity:** quarterly. |
| **EHR _7** | To incorporate social prescription in medical record (to permit linking non-medical social networks from the community to the primary care system. These networks or sources could include physical activities, learning activities, volunteerism, mutual assistance, fraternity and self-help groups, creative and arts lessons, as legal guide and support for parental problems etc.) | **Numerator:** number of patients with chronic conditions participating (during 3 months) in activities with social prescription approach.  **Denominator:** total number of patients with chronic conditions attended in the health area during the last year.  **Standard reference:** >5% in the first year, >15% in the second, >20% in the third year, >25% in the third year, >25% from the fourth year. | | | **Data source:** medical record.  **Periodicity:** semester. |
| **Organizational/procedures** | | | | | |
| **OP_1** | To ensure availability of alternatives for people with digital gap to avoid its impact on assistance quality. | **Numerator**: number of patients not included in telemedicine programs (expect those that rejected inclusion due to digital analphabetism or other reasons).  **Denominator**: number of patients included in telemedicine programs or using mHealth solutions supervised by healthcare professionals.  **Standard reference:** <2:5 in the first year, <1:10 from the third year. | | | **Data source:** medical record.  **Periodicity:** annual. |
| **OP_2** | Access to updated maps of social and health resources, specially to address the situations of most vulnerable persons. | **Numerator:** number of professional of the health area that accessed or downloaded from the intranet information about social and health resources during the last 6 months.  **Denominator:** number of active professionals in the health area during the last 6 months.  **Standard reference:** >85%. | | | **Data source:** intranet or from the digital clinical record.  **Periodicity:** semester. |
| **OP_3** | Availability of multidisciplinary intervention teams (professional teams of different disciplines that act in unexpected situations/situation of force) qualified to assist special collectives or centres in overflowed services. | **Numerator**: number of professionals involved in multidisciplinary seminaries/workshops/work to address overflowed situations (health crisis).  **Denominator**: number of active professionals in centres of the health area.  **Standard reference:** >7%. | | | **Data source:** management agreement.  **Periodicity:** annual. |
| **OP_4.1** | To extend equally the use of high-resolution consultation to polymedicated patients as in the case of those with preferential access to consultations and diagnosis tests (preferential health-card access) applying producers to ensure its correct usage. | **Numerator 1:** number of polymedicated chronic patients included in high resolution consultations procedures.  **Denominator 2:** number of polymedicated patients (with more than 5 drugs per day) attended during the last three months.  **Standard reference:** >90%. | | | **Data source:** medical record.  **Periodicity:** quarterly. |
| **OP_4.2** | To extend equally the use of high-resolution consultation patients suffering chronic conditions as in the case of those with preferential access to consultations and diagnosis tests (preferential health-card access) applying producers to ensure its correct usage | **Numerator 2:** number of patients with preferential access attended in less than 10 minutes respect the scheduled time.  **Denominator 2**: number of patients with preferential access attended in the last three months in the centre.  **Standard reference:** >90%. | | | **Data source:** medical record and consultations agenda.  **Periodicity:** quarterly. |
| **OP_5** | To measure person's experience systematically by social and health care systems, linking these results to improving measures. | **Numerator:** number of patients with positive experience with the organization and the received assistance (score higher than percentile 75 of the applied scale).  **Denominator:** number of patients that participate in patient experience analysis studies.  **Standard reference:** >85%. | | | **Data source:** management agreements.  **Periodicity:** annual. |
| **Activation of the person to be an active agent of their health** | | | | | |
| **ACTV_1** | Patients with active personalized care plan, including lifestyle information (eating habit, physical activity, sleep), impact on social and occupational activity, pharmaceutical and non-pharmaceutical treatment and comorbidities management. | **Numerator:** number of patients suffering of chronic conditions with personalized care plan established with healthcare agents.  **Denominator:** number of patients suffering of chronic condition (3 or more) attended in the health care centre.  **Standard reference:** >45% in the first year, >65% in the second, >20% in the third year, >85% from the third year. | | **Data source:** unique electronic clinical record.  **Periodicity:** quarterly | |
| **ACTV_2** | Persons with adequate adherence with the agreed therapeutic goals, for pharmaceutical and non- pharmaceutical treatment (For example, INR therapeutic goals range percentage, daily distance or steps). | **Numerator:** number of patients with adequate adherence to personalized care plan.  **Denominator:** number of patients with personalized care plan.  **Standard reference:** >75%. | | **Data source:** medical record.  **Periodicity:** semester | |
| **ACTV_3** | Complex chronic patients (2+3) with the support and intervention of nurse cases manager to increase the high-resolution consultations, to ensure coordination between specialties and another assistance levels, including psychosocial care and reducing safety incidents during referrals or transitions. | **Numerator:** number of complex chronic patients included and activated in nurse cases manager list.  **Denominator:** number of complex chronic patients (2+3) attended in the centre.  **Standard reference:** >85%. | | **Data source:** medical record.  **Periodicity:** semester | |
| **New goals** | | | | | |
| **GOAL_1** | To review procedures and resources to consider relevant relational aspects for isolated patients and their families. | **Numerator:** number of isolated patients/families with positive experience with the organization and the received assistance (score equal or higher than percentile 75 of the applied scale)  **Denominator:** number of isolated patients /families that participated in patient experience analysis studies conducted by the health area.  **Standard reference:** >85%. | **Data source:** management agreement.  **Periodicity:** annual. | | |
| **GOAL_2** | To address the low-value practice (Not-to-Do) that impact negatively on patients and the sustainability of public health system | **Numerator:** number of management agreements related to reduce low-value practices.  **Denominator:** number of management agreements objectives addressed by health care services and assistance units.  **Standard reference:** >10% in the first year, >20% from the second. | **Data source:** management agreement.  **Periodicity:** annual. | | |
| **GOAL_3** | To increase care quality establishing the quadruple approach as target. | **Numerator:** number of professionals that valued the organization positively, leadership styles and work wellbeing (score equal or higher than 75 percentile of the applied scale).  **Denominator:** number of professional in the health area.  **Standard reference:** 5 of 10 points | **Data source:** study of organizational culture and morals of the health workforce  **Periodicity:** biannual. | | |
| **GOAL_4** | To improve person’s life conditions. To reduce days of work leaves related to health condition (For example to perform a test or for medical consultations). | **Numerator:** number of patients requiring an accreditive document of diagnostic tests and consultations attendance to justify absent from work.  **Denominator:** number of patients that attended diagnostic tests or assisted in the centre.  **Standard reference:** <3%. | **Data source:** digital clinical record.  **Periodicity:** annual. | | |
| **GOAL_5** | To Incorporate STOPP-START criteria or potential inadequate drugs list in pharmacy services to avoid security incidents with medication of persons assisted in social-health centres. | **Numerator:** number of prescriptions for patients over 65 years with STOP-START criteria/ list of potential inadequate drugs.  **Denominator:** total number of prescriptions for patients over 65 years attended at the centre.  **Standard reference:** >75%. | **Data source:** pharmacy information system.  **Periodicity:** semester. | | |
